# Microhardness Recovery Kinetics, Surface Roughness Trajectories, and Erosive Wear Susceptibility of Enamel After Three In-Office Bleaching Protocols: A 28-Day In Vitro Investigation

**DOI:** 10.3390/dj14070434

**Published:** 2026-07-13

**Authors:** Berivan Laura Rebeca Buzatu, Magda Mihaela Luca, Roxana Buzatu

**Affiliations:** 1 Faculty of Dental Medicine, Doctoral School, “Victor Babeș” University of Medicine and Pharmacy Timisoara, 300041 Timisoara, Romania; berivan.buzatu@umft.ro; 2 Translational and Experimental Clinical Research Centre in Oral Health, Department of Preventive, Community Dentistry and Oral Health, “Victor Babeș” University of Medicine and Pharmacy, 300041 Timisoara, Romania; 3 Pediatric Dentistry Research Center (Pedo-Research), Department of Pediatric Dentistry, Faculty of Dental Medicine, “Victor Babes” University of Medicine and Pharmacy Timisoara, 300041 Timisoara, Romania; 4 Department of Dental Aesthetics, Faculty of Dental Medicine, “Victor Babes” University of Medicine and Pharmacy Timisoara, 300041 Timisoara, Romania; roxana.buzatu@umft.ro

**Keywords:** tooth bleaching, dental enamel, hardness, hydrogen peroxide, surface properties

## Abstract

**Background and Objectives:** In-office bleaching is one of the most requested aesthetic procedures, but its biomechanical aftermath on enamel—particularly the recovery trajectory of microhardness, surface roughness, and erosive vulnerability—remains incompletely characterised. The present study quantified the 28-day mechanical recovery profile of enamel after three commonly used chairside bleaching protocols and examined inter-relationships between mechanical, topographic and erosive endpoints. **Methods:** Forty-two human molars and premolars (27 molars, 15 premolars) extracted for clinical reasons and previously catalogued in the institutional biobank were sectioned along the cervical–occlusal axis to obtain matched control and experimental halves. The experimental halves were assigned (*n* = 14 per group) to Opalescence Quick (45% carbamide peroxide, no light), Opalescence Boost (40% hydrogen peroxide, chemically activated), or BlancOne Ultra+ (35% hydrogen peroxide with light activation). Vickers microhardness (VHN), profilometric roughness (Ra), and erosive wear after a standardised citric-acid challenge were measured at baseline, immediately post-bleaching, and at 24 h, 7 d, 14 d, and 28 d in artificial saliva. Wilcoxon signed-rank, Kruskal–Wallis with Dunn–Bonferroni post hoc, linear mixed-effects models, multiple linear regression, ROC analysis, and Spearman correlations were used (α = 0.05). **Results:** Immediate VHN drops were −13.2 ± 3.9%, −22.6 ± 3.6%, and −29.6 ± 4.7% for Opalescence Quick, Opalescence Boost, and BlancOne Ultra+, respectively. By 28 days, recovery reached 97.4 ± 2.2%, 96.0 ± 2.9%, and 91.6 ± 2.3% of baseline (*p* < 0.001). Exponential rate constants were 0.137, 0.075, and 0.092 d^−1^. Erosive wear after acid challenge was 2.1×, 2.8×, and 3.8× control values. ROC analysis identified Δ-immediate Ra as the strongest predictor of incomplete recovery (AUC = 0.859). **Conclusions:** Higher-aggression protocols delayed mechanical recovery and amplified erosive susceptibility, with light-activated systems carrying the greatest residual risk. These in vitro findings provide an evidence base for the design of future clinical studies on post-bleaching enamel recovery and remineralisation.

## 1. Introduction

Tooth bleaching is now among the most frequently requested aesthetic procedures worldwide, propelled by social-media-driven beauty standards and by an expanding portfolio of in-office and at-home whitening systems [1,2]. Within the chairside category, high-concentration hydrogen peroxide (HP) and carbamide peroxide (CP) gels remain the workhorses, producing visible whitening within a single session through the oxidative breakdown of chromogenic organic molecules embedded in the enamel matrix. Although aesthetic outcomes are well documented, the structural cost paid by enamel for that colour change is far less consistently characterised, and the interval over which any structural alteration persists has practical implications for restorative timing, dietary advice, and adjunctive remineralisation strategies [3,4].

Mechanistically, bleaching relies on the diffusion of peroxide species into enamel where reactive oxygen species (ROS) attack chromophores and, secondarily, the organic and inorganic constituents of hydroxyapatite [3]. Carbamide peroxide releases its active HP fraction more slowly than free HP and is generally considered to produce gentler kinetics, while light- or chemically activated HP gels deliver radical bursts that accelerate whitening but also raise the transient acidity and oxidative load at the gel–enamel interface [5]. These differences in radical flux, pH, and exposure time are the primary determinants of any structural footprint, far more than peroxide concentration considered in isolation [4].

Past studies have demonstrated that all three protocols produced clinically perceptible colour shifts but with markedly different magnitudes and durability of effect [6]. The second [7,8,9,10,11], leveraging scanning electron microscopy with energy-dispersive X-ray spectroscopy (SEM–EDS), revealed a severity gradient of surface and elemental alterations (BlancOne Ultra+ > Opalescence Boost > Opalescence Quick), with calcium and phosphorus depletion most pronounced in the light-activated 35% HP group. What is conspicuously missing from the literature on this cohort and on bleaching research more broadly is a longitudinal description of how the mechanical properties of bleached enamel evolve once the bleaching agent is washed away [7,12,13,14,15,16].

It is precisely this temporal dimension—how, and how completely, enamel recovers once the bleaching agent has been removed—that remains the least-resolved aspect of the field, and that motivates the present work. Comparisons of bleaching systems are by now abundant, but they overwhelmingly characterise the acute insult at a single immediate post-treatment timepoint and stop there [10,17,18,19,20,21,22]. What is known about the recovery phase is fragmentary: saliva, whether natural or artificial, can partially restore lost microhardness over a period of days to weeks, and topical fluoride or calcium–phosphate agents accelerate this rebound [23,24,25]. Several questions of direct clinical relevance, however, remain unanswered. The functional form of the recovery curve has not been formally modelled, so it is unclear whether enamel re-mineralises in a linear fashion or—as is typical of diffusion-limited biological repair—follows an exponential approach to baseline that can be summarised by a single rate constant and half-time. It is similarly unknown whether the speed and completeness of recovery differ systematically between low- and high-aggression chairside protocols, and whether any property measurable immediately after the session can forecast which specimens will ultimately recover and which will not. These gaps matter because the duration of the post-bleach vulnerability window, rather than the depth of the immediate insult alone, is what should govern the timing of adhesive restorations, the scheduling of remineralising interventions, and dietary counselling. The novelty of the present study therefore lies not in re-comparing three bleaching systems, but in resolving their 28-day recovery kinetics, fitting these kinetics to an explicit exponential model, and testing—through regression and receiver-operating-characteristic analysis—whether an early marker can predict the completeness of that recovery.

Vickers microhardness has long served as a sensitive surrogate for surface mineralisation and as an indirect marker of enamel resistance to acid challenge and abrasive wear [8,9]. A loss of microhardness immediately after bleaching has been reported in many studies, with values ranging from 8% to 30% depending on agent and protocol [10]. Critically, however, most of those reports captured a single immediate post-treatment timepoint or, at best, a single follow-up several days later. Whether the recovery follows a roughly linear remineralisation trajectory or, as kinetics-driven biological processes often do, an exponential approach to baseline characterised by a rate constant k and a half-time t½, has not been systematically described. The shape of that recovery curve has clinical consequences, because adhesive procedures, dietary chromogen exposure, and the timing of fluoride or CPP-ACP application are all decisions that hinge on how long enamel remains in a vulnerable state.

Surface roughness, captured profilometrically as Ra, and erosive wear susceptibility, quantified after a standardised acid challenge, complete the biomechanical picture [9,10]. Both can plausibly couple to bleaching-induced changes: increased roughness offers more reactive surface area for further demineralisation and biofilm retention, while reduced microhardness signals a less mineralised, more dissolution-prone surface layer [5]. The degree to which these three properties—microhardness recovery, roughness recovery, and acid-induced erosion—covary at the level of the individual specimen, and whether any single early marker can predict the longer-term recovery trajectory, would offer a clinically translatable tool but has not, to our knowledge, been examined in a multimodal manner within a single cohort.

This in vitro study therefore had three aims. First, to describe the trajectory of Vickers microhardness, profilometric Ra, and erosive wear susceptibility in enamel exposed to Opalescence Quick (45% CP), Opalescence Boost (40% HP, chemically activated), and BlancOne Ultra+ (35% HP, light-activated), over 28 days of storage in artificial saliva. Second, to fit recovery kinetics to nonlinear exponential models and derive rate constants and half-times for each protocol. Third, to examine, with multimodal correlations, multiple linear regression, and ROC analyses, whether early post-bleach markers—Δ-immediate VHN, Δ-immediate Ra, or erosive wear—can predict the eventual completeness of mechanical recovery. We hypothesised that the severity gradient already documented in the CLSM and SEM–EDS analyses (BlancOne Ultra+ > Boost > Quick) would extend into the mechanical domain and would be accompanied by slower recovery kinetics and greater residual erosive susceptibility.

## 2. Materials and Methods

### 2.1. Study Design, Ethics, and Sample Size

This was a prospective, in vitro, controlled experimental study performed at the Translational and Experimental Clinical Research Centre in Oral Health, Faculty of Dental Medicine, “Victor Babeș” University of Medicine and Pharmacy, Timișoara, Romania. All procedures were conducted in accordance with the Declaration of Helsinki and the principles of Good Practice in Biomedical Research. Ethical approval was granted by the Bioethics Committee of the “Victor Babeș” University of Medicine and Pharmacy, Timișoara (Approval No. 09/11 March 2024), and informed consent for biobanking and downstream research use had been obtained from all donors at the time of extraction.

**Figure 1:**
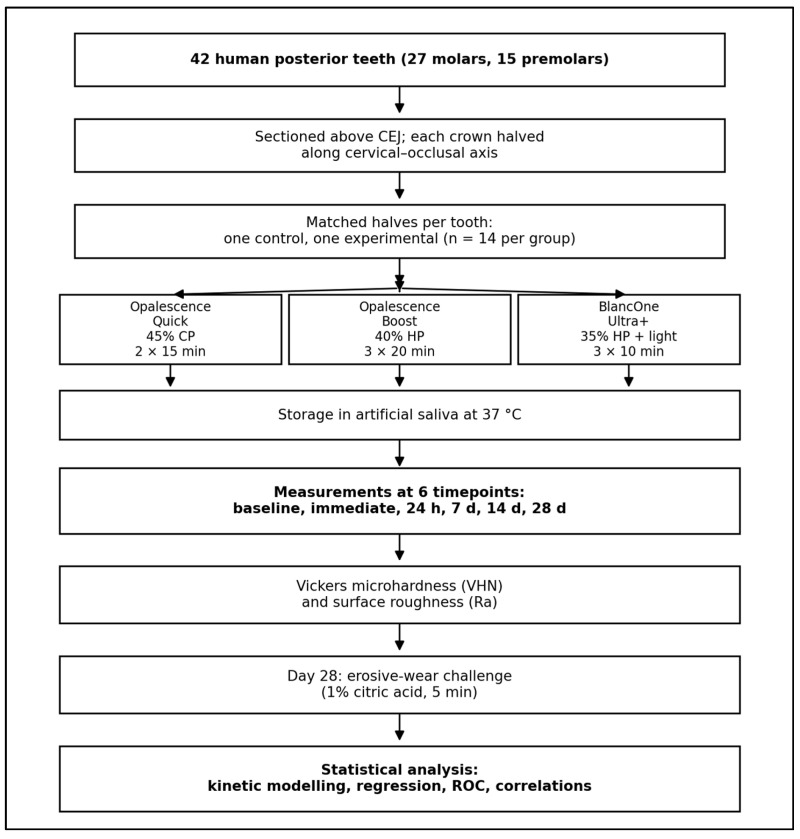
Flow diagram of the experimental design, showing specimen sourcing, within-tooth split-half allocation into matched control and experimental halves, randomisation of experimental halves to the three bleaching protocols, artificial-saliva storage, the six measurement timepoints for Vickers microhardness and surface roughness, the day-28 erosive-wear challenge, and the statistical-analysis pipeline. CEJ, cementoenamel junction; CP, carbamide peroxide; HP, hydrogen peroxide.

The required sample size was determined a priori using G*Power 3.1 [14]. For the primary outcome—between-group difference in Δ-immediate Vickers microhardness—an anticipated effect size of f = 0.45, an α of 0.05, and a power (1 − β) of 0.80 indicated that 12 specimens per group would be sufficient. To accommodate up to 20% attrition during the polishing and indentation cycles, 14 specimens per group were planned, yielding a total of 42 specimens. No specimens were lost or excluded during the present analyses; all 42 were retained through the full 28-day protocol. Tooth-type distribution per group (Opalescence Quick: 9 molars, 5 premolars; Opalescence Boost: 8 molars, 6 premolars; BlancOne Ultra+: 10 molars, 4 premolars) did not differ significantly between groups (χ^2^ = 0.622, *p* = 0.733), supporting between-group comparability for tooth-type-sensitive endpoints. The overall experimental workflow, spanning specimen sourcing through allocation, bleaching, storage, and the sequence of measurement timepoints, is summarised schematically in Figure 1.

### 2.2. Specimen Preparation and Bleaching Protocols

Teeth extracted for clinical reasons were cleaned of soft-tissue debris using periodontal curettes, stored in 0.1% thymol for five days, and screened at ×10 magnification (OMS2356, Zumax Medical, Suzhou, China) to exclude any presenting with caries, restorations, fractures, or visible enamel defects. Sectioning was performed 2 mm above the cementoenamel junction with a diamond saw under continuous water cooling, and each crown was then halved longitudinally along the cervical–occlusal axis. The two halves of every tooth shared an identification number; one was randomly designated control, the other experimental. Each half was embedded in self-cure acrylic resin (UNIFAST Trad, GC America Inc., Alsip, IL, USA), polished sequentially with Soft-Lex discs (3M ESPE) of 42, 30, and 15 μm grit for two minutes each, and rinsed with distilled water. Throughout the experiment, specimens were stored in renewed artificial saliva (formulation per Vilhena et al. [13]) at 37 °C in a biological incubator, with the medium replaced every 48 h.

The experimental halves were allocated using a stratified block randomisation procedure (Research Randomizer v4.0) to maintain comparable molar/premolar proportions across groups. Opalescence Quick (45% CP, Ultradent Products Inc., South Jordan, UT, USA) was applied as a 1–2 mm layer for two consecutive 15 min applications without light activation. Opalescence Boost (40% HP, Ultradent) was used in three consecutive 20 min chemically activated applications. BlancOne Ultra+ (35% HP, IDS S.p.A., Savona, Italy) was reconstituted from powder and liquid activator and applied in three 10 min cycles each terminated by light activation per manufacturer’s dedicated lamp. Between cycles within a session, residual gel was rinsed with distilled water and refreshed gel was applied. After the final bleaching cycle, specimens were rinsed for 60 s with distilled water, gently dried, and immediately returned to artificial saliva storage at 37 °C. For all three systems, the application time, number of cycles, and activation procedures followed the respective manufacturers’ current instructions for use without modification, and were identical to the protocols validated in our companion colour and SEM–EDS studies on the same biobank cohort [6,7]; the consolidated composition and application parameters of each product are summarised in Table 1.

### 2.3. Vickers Microhardness, Profilometric Roughness, and Erosive Wear Assessment

Surface Vickers microhardness (VHN) was measured with a microhardness tester (FM-810, Future-Tech Corp., Kawasaki, Japan) using a load of 100 gf (0.98 N) and a dwell time of 15 s, in accordance with ISO 6507-1 [11]. Five indentations were placed in the central polished region of each specimen, separated by at least three times the indentation diagonal to prevent stress-field overlap, and the average value was used as the specimen’s VHN reading at that timepoint. Indentation diagonals were measured immediately after unloading with a 40× objective and the integrated optical system, by a single trained operator (B.L.R.B.) who was blinded to group assignment by means of coded specimen labels. Intra-operator reliability over 20 duplicate readings on calibration blocks yielded an intraclass correlation coefficient of 0.97 (95% CI 0.94–0.99).

Surface roughness was quantified with a contact profilometer (Surftest SJ-310, Mitutoyo, Kawasaki, Japan) using a 2 μm diamond stylus, a measurement length of 4.0 mm, a sampling speed of 0.5 mm/s, and a Gaussian filter with a 0.8 mm cut-off in accordance with ISO 4287 [12]. Three parallel traces 0.5 mm apart were averaged to obtain the specimen Ra (arithmetic mean roughness, μm). Erosive wear susceptibility was tested only at the end of the 28-day observation period: each specimen was subjected to a standardised challenge in 1% citric acid (pH 3.0) for 5 min under constant magnetic stirring at 100 rpm, after which step-height enamel loss versus an adjacent acid-protected reference strip was measured profilometrically. The reference strip was created by masking a 1 mm wide band of the polished enamel surface with an acid-resistant adhesive polyvinyl chloride (PVC) tape before the acid challenge; after the challenge, the tape was removed, and the resulting step between the protected (baseline) and exposed (eroded) zones was used as the zero-wear datum for the profilometric step-height measurement. All measurements (VHN, Ra, erosive wear) were repeated at six timepoints: baseline (pre-bleach, on the control half mirror image), immediately after bleaching, and at 24 h, 7 days, 14 days, and 28 days of artificial-saliva storage. Between measurement sessions, specimens remained continuously in the 37 °C incubator. At each measurement session the specimens were removed from the artificial saliva, rinsed with distilled water, and gently blot-dried with absorbent paper for a standardised interval (approximately 30 s) immediately before VHN and Ra acquisition, then returned to the storage medium; the profilometer stylus contacted only the central polished window and the same operator performed all handling, so that any influence of this brief, uniform drying-and-contact cycle was applied identically to every specimen and timepoint and is therefore expected to act as a constant rather than as a source of differential bias across the roughness trajectory.

### 2.4. Statistical Analysis

Continuous variables are reported as mean ± standard deviation. Distributions were checked for normality with the Shapiro–Wilk test and visual inspection of Q–Q plots; because several outcomes failed to meet normality assumptions, non-parametric tests were used throughout for between-group and within-group comparisons. Within-group changes from baseline were tested using the Wilcoxon signed-rank test. Between-group comparisons at each timepoint were performed with the Kruskal–Wallis H test, followed by Dunn’s post hoc test with Bonferroni correction when overall significance was reached.

To capture the longitudinal trajectory of microhardness recovery, a linear mixed-effects model was fitted with VHN as the dependent variable, log-transformed time-post-bleach as a continuous predictor, group as a categorical fixed effect (with Opalescence Quick as the reference), the group × log-time interaction, and a random intercept per tooth to account for repeated measurements. Mean recovery curves were additionally fitted with a three-parameter exponential model VHN(t) = baseline − drop × exp(−k·t), and the recovery rate constant k and half-time t½ = ln(2)/k were derived for each group. Multiple linear regression was used to identify independent predictors of 28-day recovery percentage, with the absolute Δ-immediate VHN, Δ-immediate Ra, tooth type (molar vs. premolar), and group indicators as covariates. Spearman’s rank correlation coefficient (ρ) was used to examine inter-relationships between mechanical, topographic and erosive outcomes. Receiver operating characteristic (ROC) curves were constructed to assess the ability of early post-bleach markers to discriminate specimens that did versus did not achieve ≥95% recovery of baseline VHN at 28 days, with the optimal cutoff identified by Youden’s J statistic. Subgroup analyses for tooth type used Mann–Whitney U tests with effect-size estimates by Cohen’s d and Cliff’s δ (95% bootstrap confidence intervals, 2000 resamples). All analyses were performed in Python 3.12 (NumPy, SciPy 1.13, statsmodels 0.14) and SPSS v23 (IBM Corp., Armonk, NY, USA). A two-tailed α of 0.05 defined statistical significance.

## 3. Results

The results are presented in the following order: first, the baseline comparability of the three protocol groups; second, the immediate impact of bleaching on Vickers microhardness and its 28-day recovery trajectory, including the exponential kinetic modelling; third, the parallel changes in surface roughness; fourth, the erosive-wear susceptibility measured at the end of the observation period; and finally, the multimodal correlation, mixed-effects, regression, and ROC analyses that link these endpoints and identify early predictors of recovery completeness. The baseline characteristics of the study sample and the comparability of the three groups are summarised in Table 2.

Forty-two human posterior teeth (27 molars, 64.3%; 15 premolars, 35.7%) were distributed across the three protocol groups (14 per group). Mean baseline Vickers microhardness ranged from 339.7 ± 9.1 to 340.8 ± 10.3 VHN, with no statistically significant between-group difference (Kruskal–Wallis H = 0.058, *p* = 0.971). Mean baseline surface roughness Ra ranged from 0.163 ± 0.049 to 0.207 ± 0.033 μm, with a marginal between-group difference (H = 6.021, *p* = 0.049). Tooth-type distribution did not differ significantly between groups (χ^2^ = 0.622, *p* = 0.733). All 42 specimens were retained through the full 28-day protocol with no exclusions (Table 3).

**Figure 2:**
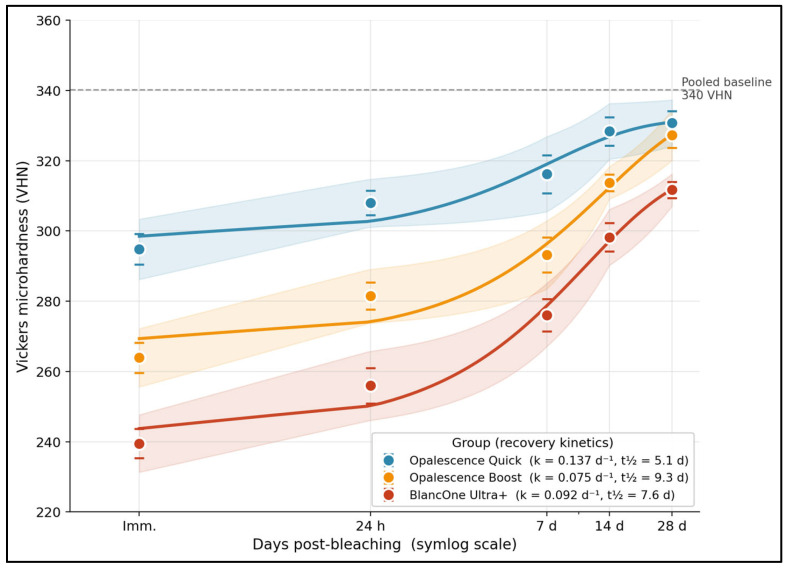
Vickers microhardness recovery trajectory of the three groups across 28 days, with exponential fits.

All three protocols produced immediate reductions in Vickers microhardness compared with baseline (Wilcoxon *p* < 0.001 in every group). Immediate post-bleach mean values were 294.8 ± 16.4 VHN (Δ% = −13.2 ± 3.9) for Opalescence Quick, 263.9 ± 15.9 VHN (Δ% = −22.6 ± 3.6) for Opalescence Boost, and 239.5 ± 15.6 VHN (Δ% = −29.6 ± 4.7) for BlancOne Ultra+. At 28 days, mean values had risen to 330.7 ± 12.6, 327.3 ± 13.6, and 311.7 ± 8.7 VHN, respectively, with Wilcoxon *p* ≤ 0.002 against baseline in every group. Between-group Kruskal–Wallis tests were significant at every post-bleach timepoint (H ranging from 15.5 to 28.3, all *p* < 0.001), as seen in Figure 2.

**Figure 3:**
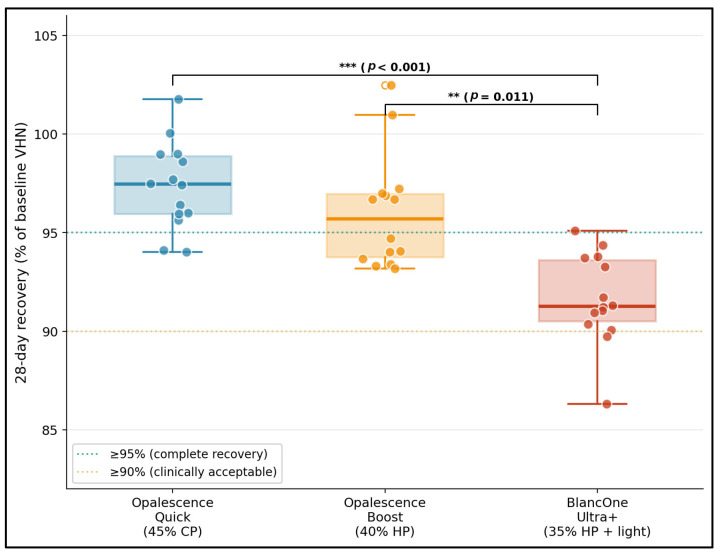
Per-specimen 28-day microhardness recovery (% of baseline VHN) by group.

Mean Vickers microhardness values (± 95% CI) are plotted across five post-bleach timepoints (immediate, 24 h, 7 d, 14 d, 28 d) on a symlog time axis, overlaid with three-parameter exponential fits VHN(t) = baseline − drop × exp(−k·t). Fit quality was R^2^ = 0.94 for Opalescence Quick, R^2^ = 0.96 for Opalescence Boost, and R^2^ = 0.98 for BlancOne Ultra+. Recovery rate constants k were 0.137 ± 0.079 d^−1^, 0.075 ± 0.043 d^−1^, and 0.092 ± 0.031 d^−1^, with corresponding half-times t½ of 5.1, 9.3, and 7.6 days. The pooled baseline of 340 VHN is shown as a horizontal dashed reference line (Table 4 and Figure 3).

Surface roughness Ra increased significantly immediately after bleaching in every group (Wilcoxon *p* < 0.001), from 0.163 to 0.244 μm in the Opalescence Quick group, from 0.207 to 0.427 μm in the Opalescence Boost group, and from 0.195 to 0.596 μm in the BlancOne Ultra+ group. At 28 days, residual ΔRa values remained statistically elevated above baseline in every group (*p* ≤ 0.020). Between-group comparison at 28 days yielded Kruskal–Wallis H = 17.53 (*p* < 0.001); on Dunn–Bonferroni post hoc testing, Opalescence Quick differed from both other groups (*p*_adj ≤ 0.011), while Opalescence Boost and BlancOne Ultra+ did not differ from each other (*p*_adj = 0.255).

Per-specimen 28-day recovery percentages are presented as jittered points overlaid on group-coloured box plots. Median recovery values were 97.4%, 95.7%, and 91.4% for Opalescence Quick, Opalescence Boost, and BlancOne Ultra+, respectively. The 95% complete-recovery and 90% clinically acceptable thresholds are shown as horizontal dotted reference lines. These two cut-offs were adopted in line with the convention in the bleaching and remineralisation literature, in which restitution of surface microhardness to within roughly 5% of pre-treatment values is treated as functionally complete recovery, while a residual deficit of up to about 10% is regarded as clinically tolerable given the natural day-to-day variability of enamel microhardness and the protective remineralising capacity of saliva [10,24,25]. The two thresholds are therefore used here as pragmatic reference benchmarks rather than as validated diagnostic limits. All 14 specimens in the Opalescence Quick group and 14 in the Opalescence Boost group exceeded the 90% threshold, whereas 7 of 14 (50%) BlancOne Ultra+ specimens did. The Kruskal–Wallis test was significant (H = 22.67, *p* < 0.001); pairwise post hoc contrasts against BlancOne Ultra+ are annotated (Table 5).

Mean enamel loss after the 5 min citric acid challenge was 1.13 ± 0.21, 1.67 ± 0.42, and 2.50 ± 0.50 μm for Opalescence Quick, Opalescence Boost, and BlancOne Ultra+, respectively, versus 0.60 ± 0.18, 0.65 ± 0.13, and 0.67 ± 0.13 μm in their matched control halves. Within-tooth Δ wear values were +0.53, +1.02, and +1.83 μm (all Wilcoxon *p* < 0.001). Bleached/control ratios were 2.13, 2.75, and 3.82. The between-bleached-groups Kruskal–Wallis test yielded H = 28.96 (*p* < 0.001); all three pairwise Dunn–Bonferroni post hoc contrasts were significant (*p*_adj ≤ 0.003), as seen in Table 6.

Mean within-tooth Δ-VHN values immediately after bleaching were −44.9 ± 13.4, −76.9 ± 11.9, and −100.7 ± 17.0 for Opalescence Quick, Opalescence Boost, and BlancOne Ultra+, respectively, and at 28 days were −8.9 ± 7.5, −13.6 ± 9.8, and −28.5 ± 8.1. Recovery percentages at 28 days were 97.4 ± 2.2%, 96.0 ± 2.9%, and 91.6 ± 2.3%. Between-group Kruskal–Wallis statistics were significant at every timepoint (H = 18.71–32.06, all *p* < 0.001). On 28-day post hoc testing, Opalescence Quick did not differ from Opalescence Boost (*p*_adj = 1.00), but both differed significantly from BlancOne Ultra+ (*p*_adj = 0.001 and *p*_adj = 0.011, respectively), as described in Table 7.

**Figure 4:**
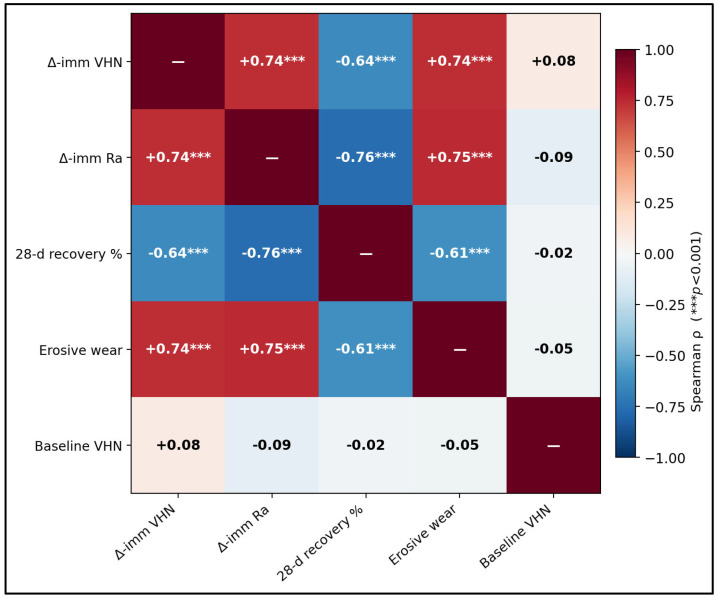
Spearman correlation heat-map of the five multimodal endpoints.

Among the four damage/recovery endpoints, every pairwise Spearman correlation was statistically significant (all *p* < 0.001), with absolute ρ values between 0.53 and 0.76. Δ-immediate |VHN| correlated positively with Δ-immediate Ra (ρ = +0.74), with erosive wear (ρ = +0.74), and with Δ-28 d VHN (ρ = +0.64). Δ-immediate Ra correlated with erosive wear (ρ = +0.75) and with ΔRa at 28 days (ρ = +0.75). The 28-day recovery percentage correlated negatively with erosive wear (ρ = −0.61). Baseline VHN showed no significant correlation with any post-bleach outcome (all *p* > 0.5), as presented in Figure 4.

Pairwise Spearman rank correlations (ρ) between Δ-immediate VHN, Δ-immediate Ra, 28-day recovery percentage, erosive wear at 28 days, and baseline VHN. Colour encodes ρ from −1 (deep blue) to +1 (deep red). Significance markers: * *p* < 0.05, ** *p* < 0.01, *** *p* < 0.001. All six pairwise correlations involving the four damage/recovery endpoints were statistically significant at *p* < 0.001, with |ρ| between 0.61 and 0.76. Correlations involving baseline VHN ranged from −0.09 to +0.08 and were not statistically significant (all *p* > 0.5), as seen in Table 8.

The mixed-effects model converged with REML estimation on 210 observations. The intercept (Opalescence Quick reference) was 297.20 ± 3.49 VHN (*p* < 0.001), and the log(time_post) main-effect slope was +10.40 ± 1.28 VHN per log-unit (*p* < 0.001). Group main effects relative to Opalescence Quick were −32.54 ± 3.92 (Opalescence Boost) and −58.02 ± 4.93 (BlancOne Ultra+), both *p* < 0.001. Group × log(time) interaction coefficients were +7.26 ± 1.81 and +10.57 ± 1.81, both *p* < 0.001. The random-intercept variance component was 62.6, and the residual variance was 179.4 (Table 9).

The model achieved R^2^ = 0.586 (Adj. R^2^ = 0.528, F(5, 36) = 10.19, *p* < 0.001), explaining recovery percentage from five candidate predictors. Of these, only Δ-immediate Ra was a statistically significant independent predictor (β = −11.57 ± 4.93, *p* = 0.024). The absolute Δ-immediate |VHN| (β = −0.010, *p* = 0.727), tooth type (β = +0.76, *p* = 0.335), and the two group indicators (p_OB = 0.667, *p*_BO = 0.532) were not retained as independent predictors. The intercept was +98.24 ± 1.59 (*p* < 0.001).

ROC curves for three candidate predictors of successful 28-day microhardness recovery (≥95% of baseline VHN), tested on 42 specimens (20 successful, 22 not successful). Area-under-the-curve (AUC) values were 0.843 for Δ-immediate |VHN| (absolute drop), 0.859 for Δ-immediate Ra, and 0.850 for erosive wear at 28 days; the corresponding Youden J statistics were 0.559, 0.618, and 0.614. Filled circles indicate the optimal Youden cutoff point for each predictor. The diagonal dashed line denotes the chance reference (AUC = 0.500).

**Figure 5:**
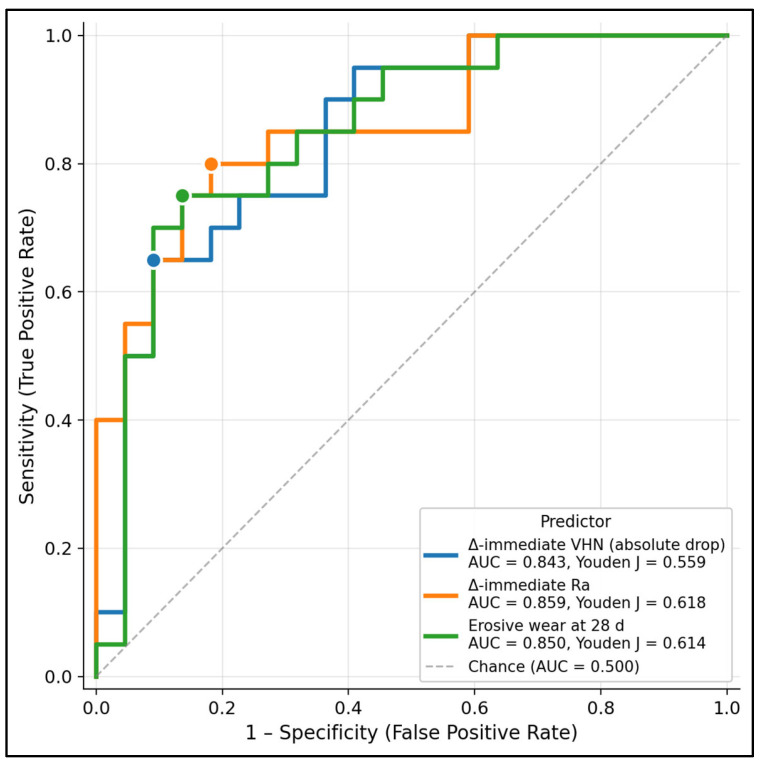
ROC curves for predicting successful 28-day microhardness recovery (≥95% of baseline VHN).

Within-group Mann–Whitney comparisons between molars and premolars did not reach statistical significance for any outcome (lowest *p* = 0.142). Effect-size estimates differed in magnitude across protocols. In the Opalescence Quick and Opalescence Boost groups, Cohen’s d ranged from −0.61 to +0.31 across the three outcomes. In the BlancOne Ultra+ group, the 28-day recovery contrast produced a large effect size (Cohen’s d = +1.01; Cliff’s δ = +0.55) favouring molars (92.3 ± 1.8% vs. 90.1 ± 2.9%); effect sizes for the other two outcomes in this group were small (d ≤ +0.23), as seen in Table 10 and Figure 5.

**Figure 6:**
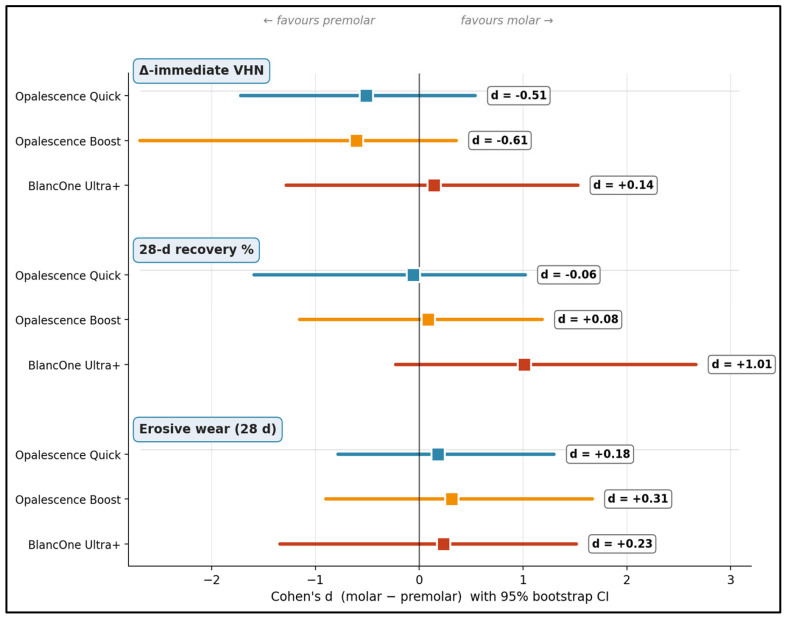
Forest plot of Cohen’s d effect sizes for molar vs. premolar comparisons within each bleaching protocol.

Cohen’s d effect sizes (squares) with 95% bootstrap confidence intervals (2000 resamples) are shown for the molar versus premolar contrast within each protocol group, separately for three outcomes: Δ-immediate VHN, 28-day recovery percentage, and erosive wear at 28 days. Positive values indicate higher mean values in molars; negative values indicate higher mean values in premolars. Effect sizes ranged from −0.61 (Opalescence Boost, Δ-immediate VHN) to +1.01 (BlancOne Ultra+, 28-day recovery %). Confidence intervals crossed zero for all nine comparisons; the BlancOne Ultra+ recovery contrast approached the zero boundary (Figure 6).

## 4. Discussion

The present study extends the multimodal characterisation of bleaching-induced enamel changes in this cohort [6,7] by quantifying, for the first time at this site, the biomechanical recovery trajectory over a 28-day post-bleach interval. The principal observation is that all three protocols produced significant acute reductions in Vickers microhardness (range −13.2% to −29.6%), followed by exponential recovery toward baseline at protocol-specific rates. The severity gradient previously established by SEM–EDS [7] (BlancOne Ultra+ > Opalescence Boost > Opalescence Quick) was reproduced in the mechanical domain, both in terms of the magnitude of the immediate insult and in terms of the residual deficit at 28 days. The narrow inter-individual variability of the recovery percentages within each group (SDs of 2.2–2.9 percentage points) suggests that the trajectory is robustly determined by protocol features rather than by inter-tooth heterogeneity.

Mechanistically, our findings cohere with the known physical chemistry of peroxide bleaching: light activation accelerates radical generation at the gel–enamel interface, transiently amplifies acidity and temperature, and produces a deeper and more disorganised surface insult [7]. The deeper structural deficit then translates into a longer recovery half-time (t½ = 7.6 days for BlancOne Ultra+ vs. 5.1 days for Opalescence Quick) and a residual erosive vulnerability that persists despite the partial restoration of surface microhardness. The negative correlation between 28-day recovery percentage and erosive wear (ρ = −0.61, p < 0.001) is biologically intuitive: enamel that re-mineralises more completely also resists subsequent acid attack better, presumably because the recovered surface is more crystalline and less porous than the disorganised post-bleach layer.

The novel translational contribution of this work lies in the predictive value of Δ-immediate Ra. In the multiple linear regression model, Δ-immediate Ra was the only statistically significant independent predictor of 28-day recovery (β = −11.57, *p* = 0.024), and in the ROC analysis, it produced the highest area under the curve (AUC = 0.859) for discriminating specimens that did and did not reach 95% recovery. It should be emphasised, however, that this predictive value was established under controlled laboratory conditions using a bench-top contact profilometer. Although intraoral and chairside roughness-assessment devices are being explored, their accuracy and reproducibility on wet, curved enamel surfaces in vivo have not yet been validated for this purpose, and the present data do not by themselves justify routine chairside profilometric screening. The Δ-immediate Ra signal is therefore best regarded, at this stage, as a candidate early marker that warrants prospective clinical evaluation rather than as a ready-to-use chairside test. The recovery rate constants reported here (k between 0.075 and 0.137 d^−1^) also provide a quantitative scaffold for designing salivary or pharmaceutical remineralisation studies, where the goal would be to demonstrate an acceleration of k rather than merely a reduction in the immediate insult. The clinical meaning of these constants is most easily appreciated through the corresponding half-times. A half-time of roughly 5 days for the carbamide peroxide protocol implies that about half of the lost surface microhardness is regained within the first working week and the great majority within two weeks, whereas the 7.6-day half-time of the light-activated 35% HP protocol implies a substantially longer interval before the surface returns to a near-baseline mineral state. If these laboratory kinetics translate even approximately to the clinic, they suggest that decisions sensitive to surface integrity should be staged against the protocol used: procedures that depend on a fully mineralised, oxygen-free enamel surface—most notably adhesive bonding of composite or veneers—would be most conservatively deferred until several half-times have elapsed (on the order of one to two weeks for the gentler protocols and longer for light-activated high-concentration HP), while remineralising measures such as topical fluoride or casein-phosphopeptide agents are likely to yield the greatest benefit when concentrated in the first post-bleach days, when the rate of natural recovery—and presumably the surface’s receptiveness to mineral uptake—is highest. By the same logic, counselling patients to limit dietary acids and chromogenic foods during this early, kinetically defined vulnerability window is a low-cost precaution that the rate constants help to time rather than leave to arbitrary convention.

Our finding of immediate Vickers microhardness reductions in the range of 13–30% places this cohort squarely within the values previously reported for in-office bleaching systems of comparable peroxide concentration. Mondelli et al. [17] reported that protocols incorporating 35–38% HP produced surface hardness reductions of 18–28% across one to three sessions, while the systematic review and meta-analysis of 55 in vitro studies by Zanolla et al. [10] concluded that low-concentration carbamide peroxide regimens (10% CP, the dominant at-home concentration) rarely cause statistically meaningful reductions in microhardness when standard application protocols are observed. The relatively gentle trajectory of our 45% CP-based Opalescence Quick group (Δ-immediate −13.2 ± 3.9%) despite its high nominal concentration reflects, in part, the slower decomposition kinetics of carbamide peroxide compared with free hydrogen peroxide. The severity gradient we observed—inversely related to the slowness of HP release kinetics—matches the morphological hierarchy documented by Spalding et al. [19] in SEM analyses of enamel exposed to 35% HP, in which the depth of structural alteration scaled with cumulative free-HP exposure rather than carbamide concentration per se. Earlier work by Tezel et al. [16] linked microhardness loss directly to detectable calcium efflux into the bleaching gel, providing a chemical correlate for the mechanical signal and complementing the EDS Ca/P depletion documented in our companion study [7]. Cavalli et al. [18] further demonstrated that fluoride or calcium addition to bleaching gels mitigates this mineral efflux without compromising whitening efficacy, supporting one of several adjunctive avenues considered below. These mechanical and chemical signals have a consistent morphological counterpart in the scanning-electron-microscopy literature. SEM studies of enamel exposed to high-concentration hydrogen peroxide have repeatedly described a shift from a smooth, intact surface to one showing shallow depressions, exposure and accentuation of the prism architecture, and a porous, honeycomb-like erosion of the interprismatic substance, with the severity of these features scaling with peroxide concentration and exposure time [19]. In our own companion SEM–EDS analysis of the same biobank cohort [7], this morphological gradient followed the identical protocol ranking observed here (BlancOne Ultra+ > Opalescence Boost > Opalescence Quick), and the most disrupted, porous surfaces coincided with the greatest calcium and phosphorus depletion. This convergence provides a structural explanation for the present quantitative findings: the increased Ra and the heightened erosive susceptibility of the light-activated group are the topographic and functional expressions of precisely the kind of porous, demineralised surface layer that SEM visualises directly, and the slower microhardness recovery of that group is consistent with the greater depth of structural reorganisation that such micrographs imply.

The mechanistic determinants of the protocol-dependent severity gradient have been increasingly clarified in recent in vitro and meta-analytic work. Jurema et al. [22] systematically varied the pH of 35% HP across acidic, neutral, and alkaline formulations and showed that, while bleaching efficacy (ΔE) was comparable across pH values, microhardness loss occurred in every formulation immediately post-bleach, suggesting that the radical flux generated by HP decomposition—rather than gel pH per se—dominates the acute mechanical insult within commonly used pH ranges. Complementary work by Ito et al. [23] demonstrated that pre-bleach pH conditioners can mitigate, though not abolish, the magnitude of subsequent hardness loss. The added contribution of light activation has been independently assessed in two influential meta-analyses. He et al. [20] aggregated 18 randomised trials and concluded that the addition of light does not enhance whitening with high-concentration HP and significantly increases the odds of post-treatment tooth sensitivity (OR ≈ 3.5 vs. non-light methods). A subsequent network meta-analysis by Maran et al. [21] ranked light-free, high-concentration HP among the most effective approaches without an added benefit from photoactivation, reinforcing the recommendation against routine light use. Our observation that BlancOne Ultra+ (35% HP with light) produced the slowest microhardness recovery (t½ = 7.6 d) and the greatest residual erosive vulnerability is consistent with these meta-analytic conclusions: the marginal pharmacokinetic advantage of photoactivation may not outweigh the deeper biomechanical insult it inflicts on enamel.

Translating the present findings into clinical guidance requires addressing three downstream concerns. First, post-bleach remineralisation strategies can substantially shorten the recovery window. Mohammadipour et al. [24] demonstrated that topical sodium fluoride applied immediately before, during, or after a bleaching session restored microhardness across multiple subsurface depths (20–120 μm) without compromising colour change, outperforming several calcium-phosphate formulations on the hardness-recovery axis. Melo et al. [25] reached a similar conclusion comparing four different remineralising agents (CPP-ACP, fluoride varnish, bioactive glass, and casein phosphopeptide combinations) following 35% HP bleaching, with most products achieving 16–33% Vickers hardness rebound within days. Borges et al. [26] further reported that the timing of remineraliser application matters, with the largest benefit accruing when application occurs in the first 60 min post-bleach. Pre-bleach toothpaste choice can also modulate the magnitude of mineral loss, as Vieira-Junior et al. [27] showed using bioactive-glass and arginine-carbonate-containing dentifrices. Second, the well-known reduction in resin–enamel bond strength immediately after bleaching, attributable to residual oxygen in the surface that inhibits free-radical polymerisation of adhesive monomers, can be reversed pharmacologically with 10% sodium ascorbate or simply by delaying adhesive procedures by one to two weeks, as established by Lai et al. [28] in the classic in vitro demonstration. Together, these strategies form an evidence-based clinical bundle that the recovery kinetics reported here can help individualise to each patient and protocol.

Two methodological choices merit explicit justification. The decision to study premolars and molars rather than the incisors on which whitening is most often judged aesthetically was driven primarily by the requirements of the within-tooth split-half design and by tissue availability. Posterior teeth, which are extracted in large numbers for orthodontic, periodontal, and surgical reasons, provided a steady supply of sound, defect-free specimens, and their broad, relatively flat buccal enamel allowed each crown to be divided into two comparable halves and to accommodate standardised microhardness indentation arrays, profilometric traces, and a masked erosive-challenge window—procedures that are far harder to standardise on the thin, strongly curved enamel of incisors. Because peroxide diffusion and the resulting oxidative chemistry act at the level of the enamel surface and are not thought to differ qualitatively between tooth types, posterior enamel is a reasonable and conservative model for the mechanical response. At the same time, anterior enamel is thinner and more curved and may behave differently in absolute terms; the present rate constants and predictive thresholds should therefore be regarded as protocol-specific and surface-level rather than as directly transferable to incisors, and confirmation in anterior teeth is an important next step. The 28-day observation window was likewise chosen deliberately. Published remineralisation studies indicate that the bulk of saliva-mediated microhardness recovery after bleaching occurs within the first one to three weeks, and our own exponential fits reached half-times of roughly 5–9 days, so that by 28 days—between three and six half-times for the three protocols—all groups had approached a plateau and the curves had become flat enough to estimate the rate constants and the asymptotic recovery percentage with confidence. Twenty-eight days therefore captures essentially the whole of the clinically relevant early recovery phase while keeping specimens viable in artificial saliva. It does not, however, exclude the possibility of slower, longer-term changes—particularly the deeper crystallographic reorganisation discussed below—and longer observation periods would be valuable for determining whether the small residual deficit seen at 28 days in the light-activated group ultimately resolves completely or persists.

This study has several limitations that should be considered when interpreting its findings. First, it is an in vitro investigation; it lacks the dynamic protective contributions of the acquired pellicle, of physiological salivary buffering with its full enzymatic and protein complement, and of normal masticatory thermomechanical cycling, all of which may amplify or mitigate the observed surface and mechanical responses under in vivo conditions. Second, the artificial saliva used for inter-session storage was renewed every 48 h according to the Vilhena protocol [13], but lacks proteinaceous and immunological constituents that modulate remineralisation kinetics; therefore, the recovery rate constants reported here may underestimate, or, in some cases, overestimate, the speed of intraoral repair. Third, the standardised citric-acid challenge we employed reflects only one of many erosive challenges described in the broader dental-erosion literature [29] and may not fully replicate the cumulative, intermittent acid exposure typical of clinical dietary patterns; the absolute erosive-wear values should therefore be interpreted comparatively rather than as predictions of clinical wear. Fourth, only molars and premolars were included, so generalisation to anterior teeth with thinner and more curved enamel should be cautious. The subgroup analysis for tooth type was underpowered to detect interactions reliably; the signal of premolar disadvantage under light-activated 35% HP warrants confirmation in a dedicated study. Finally, microhardness and Ra together capture surface mechanical and topographic responses but may miss deeper crystallographic alterations of the apatite lattice, which Bistey et al. [30] have demonstrated using FT-IR spectroscopy after HP exposure and which may evolve on a different timescale than the surface-mechanical recovery documented here [31,32,33].

## 5. Conclusions

Within the constraints of this in vitro design, all three in-office bleaching protocols caused a significant acute fall in enamel microhardness that recovered exponentially toward baseline over 28 days. The depth of the immediate insult, the speed of recovery, and the residual erosive susceptibility followed the same protocol-dependent gradient, with the light-activated 35% hydrogen peroxide system showing the largest deficit and the slowest, least complete recovery and the 45% carbamide peroxide system the smallest. Among the early markers tested, the immediate change in surface roughness was the strongest independent predictor of eventual recovery completeness. These conclusions are findings of the present laboratory model; the adjunctive measures discussed above—deferring adhesive procedures and intensifying remineralisation after bleaching—derive from previous literature rather than from this study and are noted only as context. Well-designed in vivo and clinical studies are now needed to test whether the recovery kinetics and the candidate predictive marker identified here hold under intraoral conditions.

## Figures and Tables

**Table 1:** Materials, reagents, and application parameters used in the study.

Product (Manufacturer, Location)	Type/Active Ingredient	Concentration	Application Protocol	Intended Use
Opalescence Quick (Ultradent Products Inc., South Jordan, UT, USA)	Carbamide peroxide (CP) gel, chemically buffered, no light activation	45% CP	Two consecutive 15 min applications; 1–2 mm layer; no activation	In-office/patient-assisted vital tooth whitening
Opalescence Boost (Ultradent Products Inc., South Jordan, UT, USA)	Hydrogen peroxide (HP) gel, chemically (redox) activated	40% HP	Three consecutive 20 min chemically activated applications	In-office vital tooth whitening
BlancOne Ultra+ (IDS S.p.A., Savona, Italy)	Hydrogen peroxide (HP) powder + liquid activator, light-activated	35% HP	Three 10 min cycles, each terminated by manufacturer LED light activation	In-office vital tooth whitening
Artificial saliva (formulation per Vilhena et al. [13])	Remineralising storage medium	—	Storage at 37 °C, renewed every 48 h	Specimen storage/remineralisation
Citric acid solution	Erosive challenge agent	1% (pH 3.0)	5 min immersion, magnetic stirring at 100 rpm	Standardised erosive-wear challenge
Self-cure acrylic resin (UNIFAST Trad, GC America)	Poly(methyl methacrylate) embedding resin	—	Specimen embedding	Specimen mounting

For all commercial products, the lot numbers used in this study were recorded in the laboratory logbook and are available from the corresponding author on request. Abbreviations: CP, carbamide peroxide; HP, hydrogen peroxide.

**Table 2:** Sample demographics and baseline characteristics.

Group	n	Molars	Premolars	Baseline VHN (Mean ± SD)	Baseline Ra (μm, Mean ± SD)
Opalescence Quick (45% CP)	14	9	5	339.7 ± 9.1	0.163 ± 0.049
Opalescence Boost (40% HP)	14	8	6	340.8 ± 10.3	0.207 ± 0.033
BlancOne Ultra+ (35% HP + light)	14	10	4	340.2 ± 9.2	0.195 ± 0.034
Total	42	27 (64.3%)	15 (35.7%)	—	—
Between-group p	—	χ^2^ = 0.622, *p* = 0.733	—	KW H = 0.058, *p* = 0.971	KW H = 6.021, *p* = 0.049

**Table 3:** Vickers microhardness (VHN) at each timepoint, by group; within-group Wilcoxon vs. baseline.

Group/Timepoint	VHN (Mean ± SD)	Δ% from Baseline	Wilcoxon *p*
Opalescence Quick—Baseline	339.7 ± 9.1	—	—
Opalescence Quick—Immediate	294.8 ± 16.4	−13.2 ± 3.9	<0.001
Opalescence Quick—24 h	308.0 ± 13.0	−9.3 ± 3.5	<0.001
Opalescence Quick—7 d	316.2 ± 20.4	−6.9 ± 5.0	<0.001
Opalescence Quick—14 d	328.3 ± 15.1	−3.3 ± 4.1	0.007
Opalescence Quick—28 d	330.7 ± 12.6	−2.6 ± 2.2	0.002
Opalescence Boost—Baseline	340.8 ± 10.3	—	—
Opalescence Boost—Immediate	263.9 ± 15.9	−22.6 ± 3.6	<0.001
Opalescence Boost—24 h	281.5 ± 14.6	−17.4 ± 2.7	<0.001
Opalescence Boost—7 d	293.2 ± 18.6	−14.0 ± 3.7	<0.001
Opalescence Boost—14 d	313.7 ± 8.8	−7.9 ± 2.5	<0.001
Opalescence Boost—28 d	327.3 ± 13.6	−4.0 ± 2.9	<0.001
BlancOne Ultra+—Baseline	340.2 ± 9.2	—	—
BlancOne Ultra+—Immediate	239.5 ± 15.6	−29.6 ± 4.7	<0.001
BlancOne Ultra+—24 h	256.0 ± 18.8	−24.7 ± 5.7	<0.001
BlancOne Ultra+—7 d	276.1 ± 17.2	−18.9 ± 4.3	<0.001
BlancOne Ultra+—14 d	298.2 ± 15.2	−12.3 ± 3.8	<0.001
BlancOne Ultra+—28 d	311.7 ± 8.7	−8.4 ± 2.3	<0.001

**Table 4:** Surface roughness Ra (μm) at each timepoint, by group.

Group/Timepoint	Ra (μm, Mean ± SD)	ΔRa from Baseline (μm)	Wilcoxon *p*
Opalescence Quick—Baseline	0.163 ± 0.049	—	—
Opalescence Quick—Immediate	0.244 ± 0.074	+0.081 ± 0.045	<0.001
Opalescence Quick—24 h	0.238 ± 0.068	+0.075 ± 0.050	<0.001
Opalescence Quick—7 d	0.225 ± 0.064	+0.062 ± 0.035	<0.001
Opalescence Quick—14 d	0.205 ± 0.060	+0.042 ± 0.032	<0.001
Opalescence Quick—28 d	0.201 ± 0.061	+0.038 ± 0.052	0.020
Opalescence Boost—Baseline	0.207 ± 0.033	—	—
Opalescence Boost—Immediate	0.427 ± 0.083	+0.221 ± 0.086	<0.001
Opalescence Boost—24 h	0.392 ± 0.080	+0.185 ± 0.082	<0.001
Opalescence Boost—7 d	0.380 ± 0.064	+0.173 ± 0.066	<0.001
Opalescence Boost—14 d	0.331 ± 0.060	+0.125 ± 0.057	<0.001
Opalescence Boost—28 d	0.323 ± 0.112	+0.116 ± 0.099	0.002
BlancOne Ultra+—Baseline	0.195 ± 0.034	—	—
BlancOne Ultra+—Immediate	0.596 ± 0.101	+0.400 ± 0.093	<0.001
BlancOne Ultra+—24 h	0.560 ± 0.103	+0.365 ± 0.093	<0.001
BlancOne Ultra+—7 d	0.501 ± 0.089	+0.305 ± 0.083	<0.001
BlancOne Ultra+—14 d	0.409 ± 0.063	+0.214 ± 0.050	<0.001
BlancOne Ultra+—28 d	0.390 ± 0.108	+0.195 ± 0.087	<0.001

**Table 5:** Erosive wear susceptibility (μm enamel loss) after a 5 min 1% citric acid challenge at day 28.

Group	Bleached (μm)	Control (μm)	Δ (μm)	Ratio b/c	Wilcoxon *p*
Opalescence Quick	1.13 ± 0.21	0.60 ± 0.18	+0.53 ± 0.24	2.13 ± 1.05	<0.001
Opalescence Boost	1.67 ± 0.42	0.65 ± 0.13	+1.02 ± 0.47	2.75 ± 1.20	<0.001
BlancOne Ultra+	2.50 ± 0.50	0.67 ± 0.13	+1.83 ± 0.47	3.82 ± 0.81	<0.001
KW between bleached groups	—	—	H = 28.96, *p* < 0.001	—	—

Post hoc Dunn–Bonferroni (bleached groups): Opalescence Quick vs. Opalescence Boost *p*_adj = 0.003; Opalescence Quick vs. BlancOne Ultra+ *p*_adj < 0.001; Opalescence Boost vs. BlancOne Ultra+ *p*_adj < 0.001.

**Table 6:** Δ-VHN values (from baseline) at each timepoint, with between-group Kruskal–Wallis tests.

Group/Δ-VHN	Immediate	24 h	7 d	14 d	28 d	28 d Recovery %
Opalescence Quick	−44.9 ± 13.4	−31.7 ± 12.2	−23.5 ± 17.1	−11.3 ± 14.2	−8.9 ± 7.5	97.4 ± 2.2
Opalescence Boost	−76.9 ± 11.9	−59.3 ± 9.1	−47.7 ± 12.0	−27.1 ± 8.8	−13.6 ± 9.8	96.0 ± 2.9
BlancOne Ultra+	−100.7 ± 17.0	−84.2 ± 20.2	−64.1 ± 14.7	−42.0 ± 12.9	−28.5 ± 8.1	91.6 ± 2.3
KW H	32.06	27.95	18.71	23.42	22.98	22.67
*p* value	<0.001	<0.001	<0.001	<0.001	<0.001	<0.001

**Table 7:** Spearman rank correlations between mechanical, topographic, and erosive outcomes (n = 42).

Pair	Spearman ρ	*p* Value
Δ-immediate |VHN| and Δ-immediate Ra	+0.74	<0.001
Δ-immediate |VHN| and erosive wear at 28 d	+0.74	<0.001
Δ-immediate Ra and erosive wear at 28 d	+0.75	<0.001
Δ-immediate |VHN| and Δ-28 d VHN	+0.64	<0.001
Δ-immediate Ra and ΔRa at 28 d	+0.75	<0.001
28 d recovery % and erosive wear at 28 d	−0.61	<0.001
Ra at 28 d and erosive wear at 28 d	+0.59	<0.001
VHN at 28 d and erosive wear at 28 d	−0.53	<0.001
Baseline VHN and 28 d recovery %	−0.02	0.917

**Table 8:** Linear mixed-effects model of post-bleach VHN trajectory (random intercept by tooth, REML). **Model:** VHN ~ log(time_post) + Group + log(time_post) × Group + (1 | tooth_id); reference group = Opalescence Quick.

Predictor	β (coef.)	SE	95% CI	*p* Value
Intercept	+297.20	3.49	290.36 to 304.03	<0.001
log(time_post)	+10.40	1.28	7.89 to 12.91	<0.001
Group: Opalescence Boost	−32.54	3.92	−40.23 to −24.85	<0.001
Group: BlancOne Ultra+	−58.02	4.93	−67.69 to −48.35	<0.001
log(time) × Opalescence Boost	+7.26	1.81	+3.71 to +10.81	<0.001
log(time) × BlancOne Ultra+	+10.57	1.81	+7.02 to +14.12	<0.001
Random intercept variance (tooth)	62.6	—	—	—
Residual variance	179.4	—	—	—

N observations = 210; N groups (teeth) = 28; log-likelihood = −848.6; model converged.

**Table 9:** Multiple linear regression—independent predictors of 28-day microhardness recovery (%) (n = 42).

Predictor	β	SE	t	95% CI	*p*-Value
Intercept	+98.24	1.59	61.78	95.01 to 101.46	<0.001
Δ-immediate |VHN| (absolute)	−0.010	0.027	−0.35	−0.064 to +0.045	0.727
Δ-immediate Ra	−11.57	4.93	−2.35	−21.56 to −1.59	0.024
Tooth type (molar = 1)	+0.76	0.78	0.98	−0.82 to +2.34	0.335
Group: Opalescence Boost (indicator)	+0.63	1.46	0.43	−2.33 to +3.60	0.667
Group: BlancOne Ultra+ (indicator)	−1.55	2.46	−0.63	−6.53 to +3.43	0.532
Model fit: R^2^ = 0.586, Adj. R^2^ = 0.528, F(5, 36) = 10.19, *p* < 0.001					

**Table 10:** Subgroup analysis—molar vs. premolar within each group (Mann–Whitney U with effect sizes).

Group	Outcome	Molar (Mean ± SD)	Premolar (Mean ± SD)	U	*p*	Cohen’s d	Cliff’s δ
Opalescence Quick	Δ-imm |VHN|	42.4 ± 14.0	49.3 ± 12.2	19	0.518	−0.51	−0.24
Opalescence Quick	28 d recovery %	97.3 ± 2.3	97.5 ± 2.3	20	0.606	−0.06	−0.20
Opalescence Quick	Erosive wear (μm)	1.14 ± 0.24	1.11 ± 0.16	24	0.898	+0.18	+0.07
Opalescence Boost	Δ-imm |VHN|	73.9 ± 12.9	81.0 ± 10.1	18	0.282	−0.61	−0.38
Opalescence Boost	28 d recovery %	96.1 ± 3.0	95.9 ± 3.0	27	0.755	+0.08	+0.12
Opalescence Boost	Erosive wear (μm)	1.73 ± 0.36	1.59 ± 0.51	28	0.662	+0.31	+0.17
BlancOne Ultra+	Δ-imm |VHN|	101.4 ± 16.2	98.9 ± 21.4	21	0.945	+0.14	+0.05
BlancOne Ultra+	28 d recovery %	92.3 ± 1.8	90.1 ± 2.9	30	0.142	+1.01	+0.55
BlancOne Ultra+	Erosive wear (μm)	2.53 ± 0.48	2.41 ± 0.61	21	0.945	+0.23	−0.05

## Data Availability

The data presented in this study are available on request from the corresponding author.

## References

[1] Joiner A. (2006). The bleaching of teeth: A review of the literature. J. Dent..

[2] Carey C.M. (2014). Tooth whitening: What we now know. J. Evid. Based Dent. Pract..

[3] Kwon S.R., Wertz P.W. (2015). Review of the mechanism of tooth whitening. J. Esthet. Restor. Dent..

[4] Alqahtani M.Q. (2014). Tooth-bleaching procedures and their controversial effects: A literature review. Saudi Dent. J..

[5] Dahl J.E., Pallesen U. (2003). Tooth bleaching—A critical review of the biological aspects. Crit. Rev. Oral Biol. Med..

[6] Buzatu B.L.R., Luca M.M., Galuscan A., Vaduva A.O., Fratila A.D., Dumitrescu R., Sava-Rosianu R., Balean O., Buzatu R., Jumanca D. (2025). From microstructure to shade shift: Confocal and spectrophotometric evaluation of peroxide-induced dental bleaching. J. Clin. Med..

[7] Buzatu B.L.R., Dumitrescu R., Luca M.M., Buzatu R., Galuscan A., Bolchis V., Vlase G., Vlase T., Jumanca D.E. (2025). Enamel surface and elemental changes following in vitro bleaching: A SEM-EDS approach. Dent. J..

[8] Türkün M., Sevgican F., Pehlivan Y., Aktener B.O. (2002). Effects of 10% carbamide peroxide on the enamel surface morphology: A scanning electron microscopy study. J. Esthet. Restor. Dent..

[9] Pinto C.F., Oliveira R.d., Cavalli V., Giannini M. (2004). Peroxide bleaching agent effects on enamel surface microhardness, roughness and morphology. Braz. Oral Res..

[10] Zanolla J., Marques A., da Costa D.C., de Souza A.S., Coutinho M. (2017). Influence of tooth bleaching on dental enamel microhardness: A systematic review and meta-analysis. Aust. Dent. J..

[11] (2018). Metallic Materials—Vickers Hardness Test—Part 1: Test Method.

[12] (1997). Geometrical Product Specifications (GPS)—Surface Texture: Profile Method—Terms, Definitions and Surface Texture Parameters.

[13] Abidia R.F., El-Hejazi A.A., Azam A., Al-Qhatani S., Al-Mugbel K., AlSulami M., Khan A.S. (2023). In vitro comparison of natural tooth-whitening remedies and professional tooth-whitening systems. Saudi Dent. J..

[14] Faul F., Erdfelder E., Lang A.G., Buchner A. (2007). G*Power 3: A flexible statistical power analysis program for the social, behavioral, and biomedical sciences. Behav. Res. Methods.

[15] Sharma G., Wu W., Dalal E.N. (2005). The CIEDE2000 color-difference formula: Implementation notes, supplementary test data, and mathematical observations. Color Res. Appl..

[16] Tezel H., Ertaş Ö.S., Özata F., Dalgar H., Korkut Z.O. (2007). Effect of bleaching agents on calcium loss from the enamel surface. Quintessence Int..

[17] Mondelli R.F.L., Gabriel T.R.C.G., Rizzante F.A.P., Magalhães A.C., Bombonatti J.F.S., Ishikiriama S.K. (2015). Do different bleaching protocols affect the enamel microhardness?. Eur. J. Dent..

[18] Cavalli V., Rodrigues L.K., Paes-Leme A.F., Brancalion M.L., Arruda M.A., Berger S.B., Giannini M. (2010). Effects of bleaching agents containing fluoride and calcium on human enamel. Quintessence Int..

[19] Spalding M., Taveira L.A., de Assis G.F. (2003). Scanning electron microscopy study of dental enamel surface exposed to 35% hydrogen peroxide: Alone, with saliva, and with 10% carbamide peroxide. J. Esthet. Restor. Dent..

[20] He L.B., Shao M.Y., Tan K., Xu X., Li J.Y. (2012). The effects of light on bleaching and tooth sensitivity during in-office vital bleaching: A systematic review and meta-analysis. J. Dent..

[21] Maran B.M., Ziegelmann P.K., Burey A., de Paris Matos T., Loguercio A.D., Reis A. (2019). Different light-activation systems associated with dental bleaching: A systematic review and a network meta-analysis. Clin. Oral Investig..

[22] Jurema A.L.B., de Souza M.Y., Torres C.R.G., Borges A.B., Caneppele T.M.F. (2018). Effect of pH on whitening efficacy of 35% hydrogen peroxide and enamel microhardness. J. Esthet. Restor. Dent..

[23] Ito Y., Otsuki M., Tagami J. (2019). Effect of pH conditioners on tooth bleaching. Clin. Exp. Dent. Res..

[24] Mohammadipour H.S., Shokrollahi P., Gholami S., Bagheri H., Namdar F., Sekandari S. (2024). Do different tooth bleaching–remineralizing regimens affect the bleaching effectiveness and enamel microhardness in vitro?. Int. J. Dent..

[25] Melo M., Fioresta R., Sanz J.L., Pecci-Lloret M.P., Llena C. (2022). Effect of highly concentrated bleaching gels on enamel microhardness and superficial morphology, and the recovery action of four remineralizing agents. BMC Oral Health.

[26] Borges A.B., Yui K.C.K., D’Avila T.C., Takahashi C.L., Torres C.R.G., Borges A.L.S. (2010). Influence of remineralizing gels on bleached enamel microhardness in different time intervals. Oper. Dent..

[27] Vieira-Junior W.F., Ferraz L.N., Pini N., Ambrosano G., Aguiar F., Tabchoury C., Lima D. (2018). Effect of toothpaste use against mineral loss promoted by dental bleaching. Oper. Dent..

[28] Lai S.C., Tay F.R., Cheung G.S., Mak Y.F., Carvalho R.M., Wei S.H., Toledano M., Osorio R., Pashley D.H. (2002). Reversal of compromised bonding in bleached enamel. J. Dent. Res..

[29] Lussi A., Schlüter N., Rakhmatullina E., Ganss C. (2011). Dental erosion—An overview with emphasis on chemical and histopathological aspects. Caries Res..

[30] Bistey T., Nagy I.P., Simó A., Hegedus C. (2007). In vitro FT-IR study of the effects of hydrogen peroxide on superficial tooth enamel. J. Dent..

[31] Altınışık H., Akgül S., Nezir M., Özcan S., Özyurt E. (2023). The effect of in-office bleaching with different concentrations of hydrogen peroxide on enamel color, roughness, and color stability. Materials.

[32] Salinovic I., Schauperl Z., Marcius M., Miletic I. (2021). The effects of three remineralizing agents on the microhardness and chemical composition of demineralized enamel. Materials.

[33] Irmaleny I., Hidayat O.T., Yolanda Y., Tobing E.L. (2024). Comparative evaluation of the increase in enamel hardness post-external bleaching after using casein phosphopeptide amorphous calcium phosphate fluoride (CPP-ACPF) and 5% sodium fluoride (NaF) remineralizing agents. Eur. J. Dent..

